# Epidemiology and clinical characteristics of Epstein-Barr virus infection among children in Shanghai, China, 2017-2022

**DOI:** 10.3389/fcimb.2023.1139068

**Published:** 2023-03-21

**Authors:** Zhicheng Ye, Luxi Chen, Huaqing Zhong, Lingfeng Cao, Pan Fu, Jin Xu

**Affiliations:** ^1^ Department of Clinical Laboratory, Children’s Hospital of Fudan University, National Children’s Medical Center, Shanghai, China; ^2^ Nosocomial Infection Control Department, Children’s Hospital of Fudan University, National Children’s Medical Center, Shanghai, China

**Keywords:** Epstein-Barr virus, children, coinfection, systemic lupus erythematosus, immunodeficiency, infectious mononucleosis

## Abstract

**Objective:**

To investigate the epidemiology and infectious characteristics of Epstein-Barr virus (EBV) infection among children in Shanghai, China from 2017 to 2022.

**Methods:**

We conducted a retrospective analysis of 10,260 inpatient patients who were subjected EBV nucleic acid testing from July 2017 to December 2022. Demographic information, clinical diagnosis, laboratory findings, etc. were collected and analyzed. EBV nucleic acid testing were performed by real-time PCR.

**Results:**

A total of 2192 (21.4%) inpatient children were EBV-positive, with the average age of 7.3 ± 0.1 y. EBV detection was stable from 2017 to 2020 (26.9~30.1%), but showed essential decreases in 2021 (16.0%) and 2022 (9.0%). EBV was highest (>30%) detected from three quarters (Q) including 2018-Q4, 2019-Q4 and 2020-Q3. There were 24.5% of EBV coinfection with other pathogens, including bacteria (16.8%), other viruses (7.1%) and fungi (0.7%). EBV viral loads increased when coinfecting with bacteria ((142.2 ± 40.1) ×10^4^/mL) or other viruses ((165.7 ± 37.4) ×10^4^/mL). CRP significantly increased in EBV/fungi coinfection, while procalcitonin (PCT) and IL-6 showed remarkable increases in EBV/bacteria coinfection. Most (58.9%) of EBV-associated diseases belonged to immune disorders. The primary EBV-related diseases were systemic lupus erythematosus (SLE, 16.1%), immunodeficiency (12.4%), infectious mononucleosis (IM, 10.7%), pneumonia (10.4%) and Henoch-schonlein purpura (HSP, 10.2%). EBV viral loads were highest ((233.7 ± 27.4) × 10^4^/mL) in patients with IM.

**Conclusion:**

EBV was prevalent among children in China, the viral loads increased when coinfecting with bacteria or other viruses. SLE, immunodeficiency and IM were the primary EBV-related diseases.

## Introduction

Epstein-Barr virus (EBV), also known as human herpesvirus 4, is one of the most common human viruses. EBV is highly prevalent all over the world, exposure to oral secretions has been identified as the major source for primary EBV infection in adolescents(Dunmire et al., 2018). In developed countries such as Europe and the United States, people develop primary infections in late adolescence. However, in developing areas people experience primary infection much earlier (Balfour et al., 2005).

EBV primary infection of children can result in infectious mononucleosis (IM), an acute serious condition characterized by massive lymphocytosis (Houen and Trier, 2020). Since the virus can switch between a latent and a lytic life cycle, EBV has the ability to cause chronic relapsing/reactivating infections (Murata and Tsurumi, 2014). People with weakened immune systems are more likely to develop symptoms once the virus reactivates (Kang and Kieff, 2015). Several types of cancer such as Hodgkin’s lymphoma, non-Hodgkin’s lymphoma, Burkitt’s lymphoma, and nasopharyngeal carcinoma (NC) are related to EBV infection (Gequelin et al., 2011). Moreover, chronic or recurrent EBV infection has been closely linked to some immune disorders such as systemic lupus erythematosus (SLE), systemic scleroderma (SSc), Sjögren’s syndrome (SS), rheumatoid arthritis (RA), connective tissue disease (CTD), multiple sclerosis (MS), etc. (Houen and Trier, 2020). For example, decreased immune control of chronic EBV infection has been found to be an important factor in SLE development or exacerbation (Draborg et al., 2016). Moreover, severe EBV infection are found associated with immunodeficiencies that also predispose to other viral infections and in some cases other bacterial and fungal infections (Cohen, 2015). Notably, EBV infection in individuals with primary immunodeficiencies always causes the dramatic outcomes such as severe IM, lymphoproliferation, hemophagocytic lymphohistiocytosis (HLH), and lymphoma (Tangye and Latour, 2020).

The presence of EBV in infected persons can be detected by many methods, e.g. by direct sequencing, fluorescence *in-situ* hybridization (FISH) and polymerase chain reaction (PCR) analysis of blood samples for EBV nucleic acid (Fafi-Kremer et al., 2004; Kasifoglu et al., 2018). EBV viral loads testing by quantitative DNA amplification of blood samples has proven useful for early diagnosis and monitoring post-transplant lymphoproliferative diseases (PTLD). Previous studies also suggest a role for EBV viral loads testing in nasopharyngeal carcinoma, Hodgkin’s disease, and acquired immunodeficiency syndrome (AIDS) patients with brain lymphoma, etc. (Gulley, 2001; Nadeem et al., 2021). Further research is needed to define more association of EBV infection and full spectrum of EBV-associated diseases. Moreover, the epidemiology and clinical characteristics of EBV and its associated diseases are not yet fully known among children.

Herein, we conducted a retrospective analysis on 10,260 inpatient patients who were subjected to EBV nucleic acid testing from July 2017 to December 2022, and investigated the epidemiology and infectious characteristics of pediatric EBV infection. In this study, we systematically evaluated the EBV prevalence, EBV viral loads, features of EBV co-infections, and EBV-related diseases, which can provide a comprehensive understanding of EBV infection among children in China.

## Methods

### Patients enrollment and sample collections

A total of 10,260 inpatients admitted in the Children’s Hospital of Fudan University and subjected EBV nucleic acid testing from July 2017 to December 2022. Data collection was based on the electronic medical records during hospitalization and data analysis was anonymous. Demographic information, clinical diagnosis, laboratory findings, EBV-related diseases were collected for further analysis. All experiments in the study were carried out following relevant guidelines and regulations. This study was approved by the Ethics Committee of the Children’s Hospital of Fudan University in 2021 (Approval Number: 202134).

### Plasma EBV nucleic acid testing

Peripheral blood samples (~2mL) were obtained from the admitted inpatients under aseptic operation and were collected in EDTA tube. All samples were delivered to clinical laboratory for further testing. After centrifugation, DNA was extracted from plasma samples and real-time PCR was performed (Roche LightCycler^®^ 480, Switzerland) to detect the presence and approximate amount of EBV nucleic acid in a sample. EBV Nucleic Acid Detection Kit (Real-Time PCR) produced by Daan Gene Co., Ltd, Guangzhou, China was used in this study. EBV loads test was performed according to the protocol of the commercial PCR detection kit, and a negative, critical, positive, and four standards for quantification were used in each test. DNA viral loads were calculated by comparing the cycle threshold (Ct) of the specimens to the standard curve according to the protocol.

### Laboratory testing analysis of EBV-positive cases

A series of inflammatory factors based on laboratory testing were collected and analyzed in this study, including white blood cell counts (WBC, 10^9^/L), C-reactive protein (CRP, mg/L), procalcitonin (PCT, ng/L), interleukin-6 (IL-6, pg/mL), percentage of lymphocyte (L%), percentage of neutrophil (N%) and neutrophil/lymphocyte ratio (NLR).

### EBV coinfection and EBV-related diseases screening

Single EBV infections was defined as the cases that had only EBV infections. Coinfection definitions used were those of the Centers for Disease Control and Prevention (US), which defines coinfection as one occurring concurrently with the initial infection (Feldman and Anderson, 2021). The coinfections were separated into three groups in this study: EBV/bacteria coinfection, EBV/other viruses coinfection, and EBV/fungi coinfection. EBV/other viruses coinfection was defined as EBV co-infecting with series of *respiratory virus*, *enterovirus*, *herpes simplex virus* or *cytomegalovirus* etc. EBV/bacteria or EBV/fungi coinfection was defined as EBV infection co-infecting with bacteria or fungi, consisting of those isolated from lower respiratory tracts or sterile sites including blood, cerebrospinal fluid, midstream urine etc., or enteropathogenic bacteria from stool sample. EBV/fungi coinfection was confirmed by both culture- or PCR-positive results and clinical diagnosis. Contamination microbial pathogens such as coagulase-negative *staphylococci* from non-sterile samples were excluded in this study.

The information of EBV-related diseases were obtained from the clinical diagnosis. Comparisons of clinical and infectious characteristics were conducted among patients with IM, with immune disorders and with non-immune diseases, respectively.

### Statistical analysis

Statistical analyses were performed with GraphPad Prism for Windows, version 8.00 (Graph-Pad Software). A one-way ANOVA test was used to compare the difference of laboratory testing or EBV viral loads among different groups (≥3 groups). P < 0.05 was considered statistically significant. Moreover, *t* test and Bonferroni correction were performed to compare differences of laboratory testing or EBV viral loads between two groups.

## Results

### Basic information of EBV PCR-positive cases

**Figure 1:**
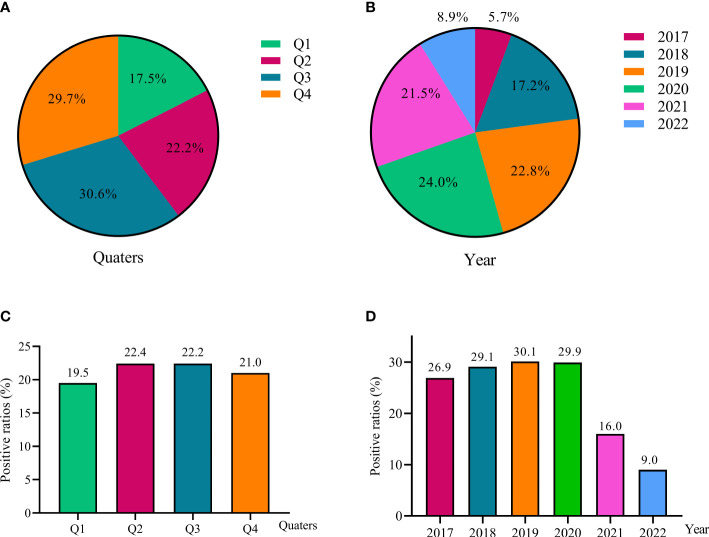
EBV detection among children from 2017 to 2022. The constituent ratios of EBV-positive cases in different quarters **(A)** and different year **(B)**; The positive ratios of EBV detection in different quarters **(C)** and different year **(D)**.

A total of 2192 (21.4%) inpatient children including 54.1% (1186) male and 45.9% female (1006) were EBV PCR-positive, and the average ages were 7.3 ± 0.1 y. The constituent ratios were 17.5%, 22.5%,30.6% and 29.7% in quarter1 (Q1), quarter2 (Q2), quarter3 (Q3) and quarter4 (Q4), respectively. EBV was highest isolated from 2019 to 2021 (21.5-24.0%), followed by 2018 (17.2%), 2017 (8.9%) and 2022 (5.7%), respectively (**Figures 1A, B**).

The EBV PCR-positive ratios were very stable from 26.9% to 30.1% during 2017 to 2020, but showed essential decreases in 2021 (16.0%) and 2022 (9.0%). There was no significant difference of EBV detections in four quarters, the ratios were ranging from 19.5% to 22.4% (**Figures 1C, D**).

**Figure 2:**
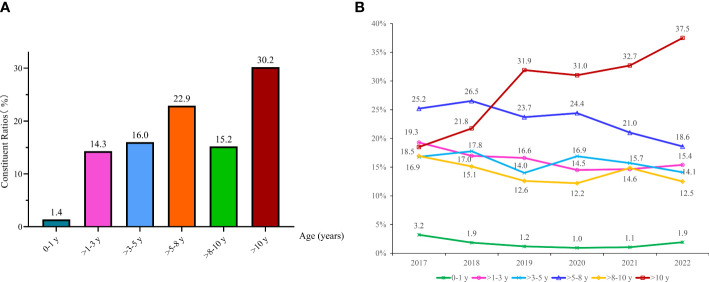
Changing of EBV detection among different age groups. The constituent ratios of EBV-positive cases in different ages **(A)**; Changing of age distributions among children from 2017 to 2022 **(B)**.

Among 2192 EBV PCR-positive cases, there were 30 infants (0-1 y, 1.4%), 313 children aged >1-3 y (14.3%), 337 children aged >3-5 y (16.0%), 482 children aged >5-8 y (22.9%), 319 children aged >8-10 y (15.2%) and 635 children aged >10 y (30.2%) (**Figure 2A**). The constituent ratios of patients aged >10 y showed a noticeable increase from 18.5% in 2017 to 37.5% in 2022, whereas the children aged >5-8 y group displayed a decrease from 2017 (25.2%) to 2022 (18.6%) (As shown in **Figure 2B**).

### Epidemiology of EBV detections in different periods

**Figure 3:**
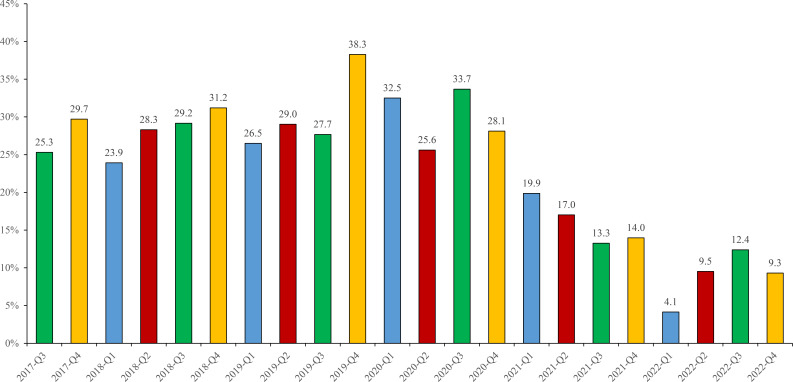
EBV-positive ratios in different periods from 2017 to 2022. Different colors represented different quarters: Q1 (blue), Q2 (red), Q3 (green), Q4 (yellow).

**Figure 3** depicted that EBV positive ratios showed a significant decrease from pre-2020 to post-2020. For example, EBV positive ratios ranged from 23.9% to 38.3% during 2017 to 2020, whereas decreased to 4.1~19.9% during 2021 to 2022.

EBV-positive cases were highest (>30%) detected from 3 periods including 2018-Q4 (31.2%), 2019-Q4 (38.3%), 2020-Q3 (33.7%), and lowest (<10%) in 2022-Q1 (4.1%), 2022-Q2 (9.5%) and 2022-Q4 (9.3%). Moreover, the highest positive ratios of EBV in each year were different, containing 2017-Q4 (29.7%), 2018-Q4 (31.2%), 2019-Q4 (38.3%), 2020-Q3 (33.7%), 2021-Q1 (19.9%), 2022-Q3 (12.4%), respectively (As shown in **Figure 3**).

### Laboratory characteristics of different type of EBV infections

Although single EBV infection is very common (1654, 75.5%), there were 368 (16.8%), 155 (7.1%) and 15 (0.7%) EBV coinfection with bacteria, other viruses and fungi, respectively. As shown in **Table 1**, the EBV viral loads varied in different types of EBV infection: compared to single EBV infection (EBV copies: (64.0 ± 11.4) × 10^4^/mL), EBV viral loads were much higher in EBV/bacteria coinfection ((142.2 ± 40.1) × 10^4^/mL) and EBV/other viruses coinfection ((165.7 ± 37.4) × 10^4^/mL) but quite lower in EBV/fungi coinfection ((1.6 ± 0.4) × 10^4^/mL, p<0.05, **Table 1**).

**Table 1:** Laboratory testing results among different types of EBV infections.

Laboratory testing	Total	Microbial infection types				*P* value
		Single EBV infection (n=1654)	EBV/bacteria coinfection (n=368)	EBV/other viruses coinfection (n=155)	EBV/fungi coinfection (n=15)	
EBV (×10^4^/mL)	83.7 ± 14.5	64.0 ± 11.4	142.2 ± 60.1	165.7 ± 77.4	1.6 ± 0.4	0.041
WBC (×10^9^/L)	9.0 ± 0.1	9.1± 0.1	9.6± 0.3	9.2 ± 0.7	7.3± 1.1	0.169
CRP (mg/L)	11.6± 0.6	11.8± 0.7	14.7± 1.6	16.0± 3.2	48.1 ± 13.5	0.036
PCT (ng/L)	0.5± 0.1	0.4 ± 0.1	1.4± 0.4	2.0± 1.4	0.3 ± 0.1	0.002
IL-6 (pg/mL)	64.8 ± 8.2	65.1 ± 9.1	132.4 ± 32.8	34.3± 7.0	175.9 ± 31.4	0.022
NLR	2.3 ± 0.1	2.3 ± 0.1	2.6± 0.2	2.4± 0.3	5.0± 0.9	0.041
N (%)	50.6± 0.4	50.6 ± 0.5	53.3 ± 1.0	47.9 ± 2.3	68.7 ± 5.6	<0.001
L (%)	38.2 ± 0.4	38.1 ± 0.4	34.8 ± 0.8	37.6 ± 2.0	21.6 ± 3.7	<0.001

White blood cell counts (WBC), C-reactive protein (CRP), Procalcitonin (PCT), Interleukin-6 (IL-6), Percentage of lymphocyte (L%), Percentage of neutrophil (N%), Neutrophil/lymphocyte ratios (NLR).

The level of WBC was normal and showed no noticeable difference among different groups, but the percentage of neutrophils (N%) and neutrophils/lymphocytes rates (NLR) were much higher in EBV/fungi coinfection (p<0.05). The average levels of CRP and PCT were normal, with a value of (11.6 ± 0.6) mg/L and (0.5 ± 0.1) ng/L respectively. However, CRP significantly increased in EBV/fungi coinfection (48.1 ± 13.5 mg/L, p<0.05), PCT showed remarkable increases in EBV/bacteria coinfection ((1.4 ± 0.4) ng/L, p<0.01) and EBV/other viruses coinfection ((2.0 ± 1.4) ng/L, p<0.01). Moreover, the level of IL-6 was increased to (64.8 ± 8.2) pg/mL and was highest in EBV/bacteria coinfection ((132.4 ± 32.8) pg/mL) and EBV/fungi coinfection ((175.9 ± 31.4) pg/mL), (p<0.05, **Table 1**).

### Characteristics of EBV-related diseases

**Figure 4:**
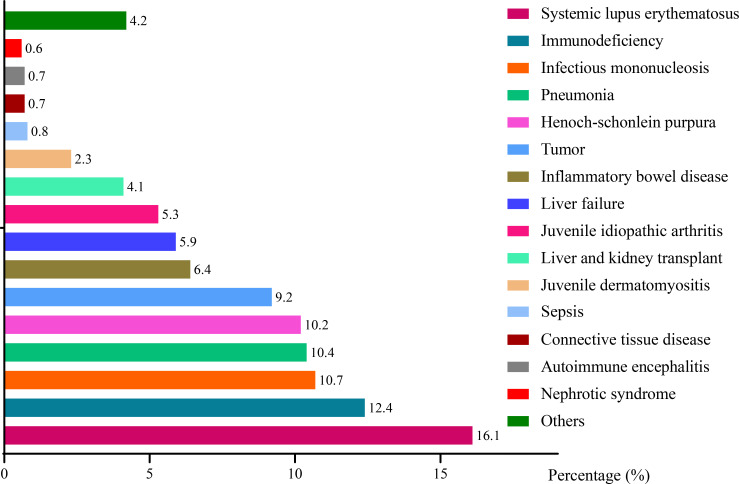
Distribution of EBV infection and its related diseases.

**Figure 4** depicted a series of EBV-associated diseases. The top-ten EBV-related diseases were systemic lupus erythematosus (SLE, 16.1%), immunodeficiency (12.4%), infectious mononucleosis (IM, 10.7%), pneumonia (10.4%), Henoch-schonlein purpura (HSP, 10.2%), tumor (9.2%), inflammatory bowel disease (IBD, 6.4%), liver failure (5.9%), juvenile idiopathic arthritis (JIA, 5.3%), liver and kidney transplant (4.1%). Other rare diseases such as juvenile dermatomyositis (JD), sepsis, connective tissue disease (CTD), autoimmune encephalitis (AE) and nephrotic syndrome (NS) took up to 2.3%, 0.8%, 0.7%, 0.7% and 0.6%, respectively. Other EBV-related diseases included series of non-immune diseases such as congenital heart disease, indigestion, digestive system diseases and rhinitis, accounting for 4.2% totally. Most of EBV-related diseases belong to immune disorders (1290, 58.9%), among which SLE was the primary one (352, 27.3%). IM and non-immune diseases including pneumonia, tumor, liver failure, sepsis etc. accounted for 10.7% and 30.5%, respectively.

**Figure 5:**
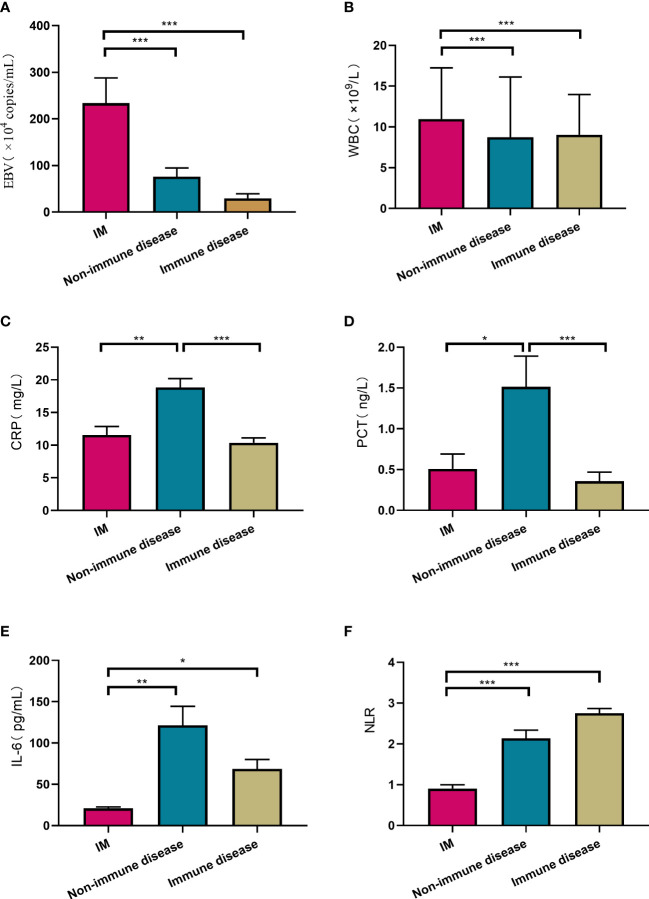
Comparison of laboratory testing results among IM, non-immune diseases and immune diseases. Laboratory testing included EBV viral loads **(A)**, WBC **(B)**, CRP **(C)**, PCT **(D)**, IL-6 **(E)**, NLR **(F)**. ^*^P<0.05, ^**^p<0.01, ^***^p<0.001. White blood cell counts (WBC), C-reactive protein (CRP), Procalcitonin (PCT), Interleukin-6 (IL-6), Neutrophil/lymphocyte ratios (NLR).

EBV viral loads were highest in IM group with a value of (233.7 ± 27.4) × 10^4^/mL, and lower in non-immune diseases ((75.9 ± 18.8) × 10^4^/mL) and immune disorder group ((29.6 ± 9.6) × 10^4^/mL), (p<0.001). Many inflammatory factors varied in different groups. For instance, the value of WBC and percentage of lymphocyte (L%) in patients diagnosed with IM was ((11.0 ± 0.4) × 10^9^/L) and (52.5 ± 1.1) % respectively, much higher than other two groups (p<0.001). CRP, PCT and IL-6 all showed significant increases in non-immune diseases than IM and immune diseases, with the value of 18.8 ± 1.4 mg/L (p<0.001), 1.5 ± 0.4ng/L (, p=0.001), and 121.4 ± 22.5pg/mL (, p<0.01), respectively. Shown in **Figure 5** and **Supplementary Table S1**.

## Discussion

EBV infects 95% of the human population and usually is asymptomatic, or in the case of adolescents and young adults can result in IM with fever, sore throat, lymphadenopathy, and splenomegaly (Cohen, 2015). EBV infection more frequently results in in a latent infection that linked with many human diseases, such as autoimmune diseases, immunodeficiency or some cancers (Jog and James, 2020; Lino and Ghosh, 2021; Patel et al., 2022). In this study, we collected a total of 2192 EBV-positive cases, systematically analyzed the epidemiology and characteristics of pediatric EBV infection. EBV was prevalent among children in China, the viral loads increased when coinfecting with bacteria or other viruses. SLE, Immunodeficiency and IM were the primary EBV-related diseases.

EBV was detected from 21.4% of pediatric patients, the positive ratios in 2021-2022 (9.0~16.0%) were much lower than that in 2017-2020 (26.9~30.1%). The lower EBV detection after 2020 might link to some factors, such as the strategies of Chinse government towards SARS-CoV2 (Severe Acute Respiratory Syndrome Coronavirus 2) including lock-down, usage of masks and keeping distances after 2020 COVID-19 (Coronavirus disease-19) year. The microbial spectrum changed dramatically during COVID-19 pandemic year, the primary respiratory bacteria including *S. pneumoniae* (Spn), *H. influenzae* (Hin), and *S. pyogenes* (Spy) and series of viruses containing *Respiratory syncytial virus* (RSV), *Influenza virus* (IV), *Metapneumovirus* (MP), *Parainfluenza viruses* (PV), *Adenovirus* (AV), *Bocavirus* (BV) and *Enterovirus* (EV) etc. have been reported to reduce remarkably during the COVID-19 pandemic year (Di Mattia et al., 2021; Fu et al., 2021; Tang et al., 2021). Herein, we also confirmed a significant reduction of EBV infection among children after COVID-19 year.

Previous study showed that EBV positive rate was the lowest at 0-1 year, then increased gradually in the older groups, EBV seroprevalence became more than 50% before age 3 in Chinese children (Xiong et al., 2014; Shi et al., 2022). Our study also confirmed that the constituent ratios of EBV infection was lowest in infants aged no more than 1 year old. Primary EBV infection has been considered common in younger children, and mainly occurred in Chinese children at 2-4 years old (Garcia-Peris et al., 2019; Shi et al., 2022). In this study, EBV ratios among children aged >10 y showed a noticeable increase from 2017 to 2022, whereas the ratios of patients aged 3-5 y was reduced slightly during 2017 to 2022. The rate and timing of EBV infections varied in different regions or countries. For instance, most children in the developing world acquire EBV infection during childhood, in contrast to most developed countries where a majority of EBV infections occur at a later age, often in adolescence (Jayasooriya et al., 2015). Our finding indicated that EBV infection became more prevalent among older children and adolescence from 2017 to 2022 in Shanghai, China.

There is a high incidence of multi-pathogen infections among human admitted with EBV infections that often develop severe and life-threatening conditions (Wen et al., 2022). Although single EBV was very common in our study, coinfections with bacteria, other virus and fungi took up to 24.5% in total. More importantly, EBV viral loads significantly increased in EBV/bacteria and EBV/other viruses coinfection. Meanwhile, many inflammatory factors were increased in EBV coinfection groups: CRP was highest in EBV/fungi coinfection, while PCT and IL-6 were increased significantly in EBV/bacteria coinfection, indicating more complicated syndromes in EBV coinfections. Therefore, coinfections of EBV cannot be ignored because it presented higher level of inflammatory factors and might cause server complications of EBV infection among children.

Although a majority of EBV infectious cases is non-complicated and may even go unnoticed (Houen and Trier, 2020), previous researchers have demonstrated that latent EBV infection was associated with a variety of diseases such as IM, immunodeficiency and autoimmune diseases, respiratory tract infection, nasopharyngeal carcinoma, etc. (Fugl and Andersen, 2019; Gao et al., 2011; Fujiwara and Nakamura, 2020; Sollid, 2022). In this study, EBV infection was observed in a series of diseases, the primary EBV-linked diseases were SLE, immunodeficiency, IM, pneumoniae and HSP. Patients with certain genetic defects of their immune system always had difficulties controlling EBV, which might cause EBV chronic infection. The mechanisms between EBV and autoimmune diseases might associate with EBV protein EBNA2. EBNA2 and its associated human transcription factors (TFs) occupy a significant fraction of autoimmune risk loci, revealing the potential mechanism how EBV associated with a series of autoimmunity (Harley et al., 2018). EBV reactivation or latent infection were associated with severe pathologies that can have fatal outcome (Worth et al., 2016; Lino and Ghosh, 2021). In this study, more than half of EBV-linked diseases belonged to autoimmune diseases or immunodeficiency, indicating a very close linkage between EBV infection and immune diseases. Moreover, EBV viral loads varied in different groups and were highest among patients with IM.

In conclusion, EBV infection are prevalent among children in Shanghai, China. EBV detection significantly reduced after COVID-19 pandemic. Coinfections of EBV to other microbial pathogens are not rare, and presented more EBV viral loads and higher level of inflammatory factors. SLE, immunodeficiency and IM are the most frequent EBV-related diseases, indicating surveillance of EBV infection are thus required among children with immune diseases.

## Data availability statement

The raw data supporting the conclusions of this article will be made available by the authors, without undue reservation.

## Ethics statement

The studies involving human participants were reviewed and approved by the Ethics Committee of the Children’s Hospital of Fudan University (ethical approval number:2021-34). Written informed consent to participate in this study was provided by the participants’ legal guardian/next of kin.

## Author contributions

JX and PF designed the experiments and revised the manuscript. PF and ZY analyzed the data and wrote the manuscript. LXC, HZ, LFC participated in the experiments and data collection. All authors contributed to the article and approved the submitted version.
